# Hemorrhage in pheochromocytoma surgery: evaluation of preoperative risk factors

**DOI:** 10.1007/s12020-021-02964-y

**Published:** 2022-04-15

**Authors:** Ying Guo, Hai Li, Dingxiang Xie, Lili You, Li Yan, Yanbing Li, Shaoling Zhang

**Affiliations:** 1 Department of Endocrinology, Sun Yat-sen Memorial Hospital, Sun Yat-sen University, Guangzhou, China; 2 Department of Endocrinology, The First Affiliated Hospital, Sun Yat-sen University, Guangzhou, China; 3 Department of Radiology, The First Affiliated Hospital, Sun Yat-sen University, Guangzhou, China

**Keywords:** Pheochromocytoma, Hemorrhage, Surgery, Risk factor, Preoperative assessment

## Abstract

**Objective:**

Pheochromocytoma surgery carries a higher risk of hemorrhage. Our objective was to identify preoperative risk factors for hemorrhage during pheochromocytoma surgery.

**Methods:**

Patients who underwent surgery and with postoperative pathological confirmation were enrolled. A total of 251 patients from our center were included in the investigation, and 120 patients from the First Affiliated Hospital, Sun Yat-sen University were included as an external validation dataset. Family and medical history, demographics, hemodynamics, biochemical parameters, image data, anesthesia and operation records, postoperative outcomes were collected. Postoperative complications were graded by the Clavien–Dindo classification. Correlation between intraoperative hemorrhage volume and postoperative outcomes was assessed. The features associated with intraoperative hemorrhage were identified by linear regression. All features that were statistically significant in the multiple linear regression were then used to construct models and nomograms for predicting intraoperative hemorrhage. The constructed models were evaluated by Akaike Information Criterion. Finally, internal and external validations were carried out by tenfold cross-validation.

**Results:**

Intraoperative hemorrhage volume was positively correlated with the postoperative hospitalization time (*R* = 0.454, *P* < 0.001) and the Clavien–Dindo grades (*R* = 0.664, *P* < 0.001). Features associated with intraoperative hemorrhage were male gender (*β* = 0.533, OR = 1.722, *P* = 0.002), tumor diameter (*β* = 0.027, OR = 1.027, *P* < 0.001), preoperative CCB use (*β* = 0.318, OR = 1.308, *P* = 0.123) and open surgery (*β* = 1.175, OR = 3.234, *P* < 0.001). Validations showed reliable results (internal (*R* = 0.612, RMSE = 1.355, MAE = 1.111); external (*R* = 0.585, RMSE = 1.398, MAE = 0.964)).

**Conclusion:**

More intraoperative hemorrhage is correlated with longer postoperative hospitalization time and more severe postoperative complications. Male gender, larger tumor, preoperative CCB use and open surgery are preoperative risk factors for hemorrhage in PCC surgery.

## Introduction

Pheochromocytoma (PCC) is an endocrine active tumor that produces catecholamines and has a risk of malignancy [1, 2].

Surgical resection is the treatment of choice. Although surgical mortality has been greatly reduced, PCC surgery carries potential risks in the tumor removal [3–5]. Due to activated angiogenesis and catecholamine release by the PCC, intraoperative hemorrhage control and maintenance of hemodynamic stability are the keys to surgical safety [3–6]. Currently, no universal method for assessing preoperative risks for PCC surgery exists. Clinicians have found some predictors of intraoperative hemodynamic conditions [7–9], but only a few investigations have reported the influencing factors of intraoperative hemorrhage [10].

Here we report the results of a study on the preoperative assessment of hemorrhage in PCC surgery. We analyzed and validated the effects of clinical features on intraoperative hemorrhage volume in PCC. Our findings might help to improve the individualized preoperative regimens of PCC surgery.

## Material and methods

### Subjects

Patients with postoperative pathology [11] confirmed between January 2001 and December 2020 in Sun Yat-sen Memorial Hospital, Sun Yat-sen University, and between January 2010 and December 2019 in the First Affiliated Hospital, Sun Yat-sen University, were enrolled. Patients from the First Affiliated Hospital served as an external validation dataset. The criteria for exclusion were as follows: (1) bilateral surgery; (2) resection involving nontumor adjacent organs; (3) inadequate medical records.

### Operative details

All the procedures were executed under general anesthesia. Furthermore, all surgeries were conducted by dedicated surgical team. Two chief surgeons were involved in the team, both of whom had over 10 years’ experience in urologic surgery.

### Data collection

We collected family and medical history, demographics, hemodynamics, biochemical parameters, image data, anesthesia and operation records, postoperative outcomes.

Blood cell counts were determined by an automated blood cell counter (Sysmex XE-2100). Plasma and urinary catecholamines were detected by radioimmunoassay methods (ALPCO, Salem, NH, USA). Plasma glucose concentration was measured by an automatic biochemical analyzer (Mindray BC-31s). Pathoglycemia was defined to include impaired fasting glucose, impaired glucose tolerance and diabetes [12]. Tumor size was defined as the maximum tumor diameter. Starting 3 days before the surgery, blood pressure (BP) was measured every 6 h. The heart rate (HR) and BP measured in the morning of the surgery were defined as the preinduction HR and BP. All HRs and BPs were measured after 15 min of rest while seated. Smoking, exercise, eating and caffeine were not allowed for 1 h prior to measurements. BP fluctuations were quantified by the maximum minus minimum BP. The volume of hemorrhage was estimated during the surgery. Volume of intraoperative hemorrhage = (intraoperative amount of liquid absorbed by the gauze + amount of liquid sucked by the equipment + amount of liquid on the wound surface) − amount of washing liquid. Postoperative complications were graded by the Clavien–Dindo classification [9].

### Preoperative management

Phenoxybenzamine (PBZ) was prescribed as a preoperative medication and was adjusted according to BP and tolerability. Calcium channel blockers (CCBs) were administered if a tolerable/sufficient dose of PBZ treatment did not achieve normotension. Treatment of tachycardia with β receptor blockers was provided if necessary. High dietary sodium and fluid intake were recommended [11].

### Statistics

RStudio software was used to perform statistical analyses. All continuous variables are presented as mean ± SD for normally distributed data or median (interquartile range, IQR) for skewed distributed data. Differences between the groups were compared by Student’s *t* test or the Mann–Whitney *U* test, as appropriate. Categorical variables are presented as frequency (percentage) and were compared using the chi-square test. Correlation coefficients were calculated between estimated intraoperative hemorrhage volume and postoperative outcomes. All the clinical features were chosen by linear regression analysis. Briefly, features with a correlation coefficient (*R*) > 0.15 and *P* < 0.10 in the intragroup comparison incorporated the initial multivariate linear regression model and then applied the MASS package of the R language to automatically filter the features by the Akaike information criterion (AIC) to derive the final multivariate linear regression model. The nomogram was formulated based on the results of the final multivariate linear regression model using the RMS package of the R language. Finally, internal and external validation were carried out by tenfold cross validation, and the *R* value, root mean squared error (RMSE) and mean absolute error (MAE) were calculated. All tests were two-tailed, and *P* < 0.05 was considered statistically significant.

## Results

### General characteristics of the subjects

The characteristics of all the patients are shown in Table 1. A total of 251 patients who underwent PCC surgery in our center participated in this study.

**Table 1:** General characteristics of patients who underwent pheochromocytoma surgery

Variables	All patients (*N* = 251)
Male, *N* (%)	114 (45.40%)
Age, years	45.42 ± 15.21
BMI, kg/m^2^	21.84 ± 2.71
Tumor Diameter, mm	52.39 ± 26.80
Family History, *N* (%)	9 (3.60%)
Hypertension, *N* (%)	204 (81.30%)
Positive Symptom, *N* (%)	179 (71.30%)
Pathoglycemia, *N* (%)	128 (51.00%)
Preoperative SBP Fluctuation, mmHg	31.87 ± 14.51
Preoperative DBP Fluctuation, mmHg	21.18 ± 9.24
Preinduction SBP, mmHg	129.54 ± 18.65
Preinduction DBP, mmHg	79.50 ± 12.96
Preinduction HR, bpm	82.98 ± 12.81
Elevated Catecholamines, *N* (%)	208 (82.90%)
RBC Count, ^10^12^/L	4.47 ± 0.68
Hb, g/L	125.85 ± 17.36
HCT	0.38 ± 0.05

*BMI* body mass index, *SBP* systolic blood pressure, *DBP* diastolic blood pressure, *HR* heart rate, *RBC* red blood cell, *Hb* hemoglobin, *HCT* hematocrit

The mean age was 45.42 ± 15.21 years old. The mean body mass index (BMI) was 21.84 ± 2.71 kg/m^2^. The most frequent manifestation was hypertension (204 cases, 81.30%). Other manifestations, such as palpitation and headache, were also common (179 cases, 71.30%). The mean PCC diameter was 52.39 ± 26.80 mm. There were 128 (51.00%) patients suffering from pathoglycemia. Elevated levels of catecholamines were found in 208 (82.90%) patients. The mean red blood cell (RBC) count was (4.47 ± 0.68) × 10^12^, the mean hemoglobin was 125.85 ± 17.36 g/L, and the mean hematocrit was 0.38 ± 0.05.

The mean preoperative systolic blood pressure (SBP) fluctuation was 31.87 ± 14.51 mmHg, and the mean preoperative diastolic blood pressure (DBP) fluctuation was 21.18 ± 9.24 mmHg. The mean preinduction SBP was 129.54 ± 18.65 mmHg, and the mean preinduction DBP was 79.50 ± 12.96 mmHg. The mean preinduction HR was 82.98 ± 12.81 bpm.

### Characteristics associated with surgery

Preoperative medical management and characteristics associated with surgery were summarized in Table 2.

**Table 2:** Characteristics associated with surgery

Variables	All patients (*N* = 251)
Preoperative CCB use, *N* (%)	58 (23.10%)
Preoperative PBZ use, *N* (%)	235 (93.60%)
Preoperative β-blocker use, *N* (%)	114 (45.40%)
PBZ treatment duration, day	17.00 (13.00, 22.00)
β-blocker treatment duration, day	14.00 (0.00, 56.00)
CCB treatment duration, day	14.00 (0.00, 44.00)
ASA score, *N* (%)	
II	56 (22.30%)
III	183 (72.90%)
IV	12 (4.80%)
Surgical approach, LA vs. open	
LA, *N* (%)	206 (82.10%)
Open, *N* (%)	45 (17.90%)
Estimated Intraoperative Hemorrhage, ml	50.00 (20.00,312.50)
Operative time, min	120.00 (90.00, 190.00)
Postoperative Complications, *N* (%)	70 (27.89%)
Clavien–Dindo I, *N* (%)	20 (7.97%)
Clavien–Dindo II, *N* (%)	40 (15.94%)
Clavien–Dindo III, *N* (%)	2 (0.80%)
Clavien–Dindo IV, *N* (%)	8 (3.19%)
ICU admission, *N* (%)	8 (3.19%)
Postoperative Hospitalization Time, day	7.00 (6.00, 10.00)

*CCB* calcium channel blocker, *PBZ* phenoxybenzamine, *ASA* American Society of Anesthesiologists, *LA* laparoscope adrenalectomy, *ICU* intensive care unit

PBZ was prescribed to 235 (93.60%) patients as a preoperative medication, while the other 16 did not take PBZ. β receptor blockers were given to 114 patients (45.40%), while 58 patients (23.10%) were prescribed CCBs. The median preoperative treatment duration of PBZ was 17.00 (13.00, 22.00) days, the median preoperative duration of β receptor blockers was 14.00 (0.00, 56.00) days, and the median preoperative duration of CCBs was 14.00 (0.00, 44.00) days.

Most patients (239/251, 95.20%) were classified as American Society of Anesthesiologists (ASA) physical status II-III. Laparoscopic adrenalectomy was performed in 206 (82.10%) patients. The median estimated intraoperative hemorrhage volume was 50.00 ml (20.00, 312.50), and the median operative time was 120 min (90.00, 190.00).

No mortality was observed within 30 days after surgery, while about a quarter of patients (70/251, 27.89%) were diagnosed with postoperative complications. Only 10 patients (3.98%) suffered from major complications (Clavien–Dindo ≥3) [9]: among them, nine presented heart failure and one suffered an ischemic stroke. Eight (3.19%) of them required aggressive management in intensive care unit. The median postoperative hospitalization time was 7 days (6.00, 10.00).

### Comparison of operative and postoperative outcomes by experience

All operations were sorted in chronological order, with the first 125 defined as less-experienced cases and the latter 126 defined as more-experienced cases. As shown in Table 3, no difference was observed between the less-experienced group and the more-experienced group when comparing the estimated intraoperative hemorrhage volume (*P* = 0.060) or the incidence of postoperative complications (*P* = 0.421). However, less-experienced group endured longer operative time (145.00 (100.00, 219.00) vs. 120.00 (90.00, 172.50) min, *P* = 0.003) and postoperative hospitalization time (8.00 (7.00, 10.50) vs. 7.00 (5.00, 8.00) days, *P* < 0.001).

**Table 3:** Comparison of operative and postoperative outcomes by experience

	All patients *N* = 251	Less-experienced *N* = 125	More-experienced *N* = 126	*P* value
Estimated intraoperative hemorrhage, ml	50.00 (20.00, 312.50)	50.00 (20.00, 400.00)	50.00 (20.00, 250.00)	0.060
Operative time, min	120.00 (90.00, 190.00)	145.00 (100.00, 219.00)	120.00 (90.00, 172.50)	0.003
Postoperative complications, *N* (%)	70 (27.89%)	32 (25.60%)	38 (30.16%)	0.421
Clavien–Dindo I, *N* (%)	20 (7.97%)	14 (11.20%)	6 (4.76%)	0.051
Clavien–Dindo II, *N* (%)	40 (15.94%)	16 (12.80%)	24 (19.05%)	
Clavien–Dindo III, *N* (%)	2 (0.80%)	1 (0.80%)	1 (0.79%)	
Clavien–Dindo IV, *N* (%)	8 (3.19%)	1 (0.80%)	7 (5.56%)	
Postoperative hospitalization time, day	7.00 (6.00, 10.00)	8.00 (7.00, 10.50)	7.00 (5.00, 8.00)	<0.001

### Intraoperative hemorrhage was correlated with postoperative outcomes

We then investigated the relationship between estimated intraoperative hemorrhage volume and postoperative outcomes. The results provided a positive correlation between the estimated intraoperative hemorrhage volume and the postoperative hospitalization time (*R* = 0.454, *P* < 0.001), and a positive correlation between the estimated intraoperative hemorrhage volume and the Clavien–Dindo grades (*R* = 0.664, *P* < 0.001), as presented in Supplementary Table 1.

### Model construction for intraoperative hemorrhage assessment and validation

Linear regression analysis determined the features correlated with estimated intraoperative hemorrhage volume as a continuous dependent variable: including male gender (*R* = 0.149, 95% CI 0.026–0.268, *P* = 0.018), tumor diameter (*R* = 0.526, 95% CI 0.430–0.610, *P* < 0.001), preoperative CCB use (*R* = 0.152, 95% CI 0.029–0.271, *P* = 0.016), open surgery (*R* = 0.424, 95% CI 0.317–0.520, *P* < 0.001) and preoperative SBP fluctuation (*R* = 0.171, 95% CI 0.048–0.289, *P* = 0.007) (Table 4). No correlation was detected between age, medical history, BMI, blood parameters, biochemical results, preoperative use of PBZ, preoperative duration of PBZ, preoperative use of *β* receptor blockers, preoperative duration of β receptor blockers, preoperative duration of CCBs, preoperative DBP fluctuation, preinduction BP, preinduction HR, ASA score and estimated intraoperative hemorrhage volume (Supplementary Table 2).

**Table 4:** Assessment model of intraoperative hemorrhage

Intercept and variable	Correlation analysis			Model 1			Model 2		
	*R*	95% CI	*P* value	*β*	Std	*P* value	*β*	Std	*P* value
Intercept	–	–	–	2.129	0.263	<0.001	2.301	0.208	<0.001
Male	0.149	0.026–0.268	0.018	0.544	0.173	0.002	0.533	0.173	0.002
Tumor Diameter	0.526	0.430–0.610	<0.001	0.027	0.003	<0.001	0.027	0.003	<0.001
Preoperative CCB Use	0.152	0.029–0.271	0.016	0.269	0.210	0.203	0.318	0.205	0.123
Surgical Procedure (Open vs. LA)	0.424	0.317–0.520	<0.001	1.174	0.239	<0.001	1.175	0.239	<0.001
Preoperative SBP Fluctuation	0.171	0.048–0.289	0.007	0.007	0.006	0.282	–	–	–
AIC	–			158.72			157.91		

*R* correlation coefficient, *β* regression coefficient, *Std* standard deviation, *OR* odd ratio, *CI* confidence interval, *CCB* calcium channel blocker, *LA* laparoscope adrenalectomy, *AIC* akaike information criterion

**Fig. 1:**
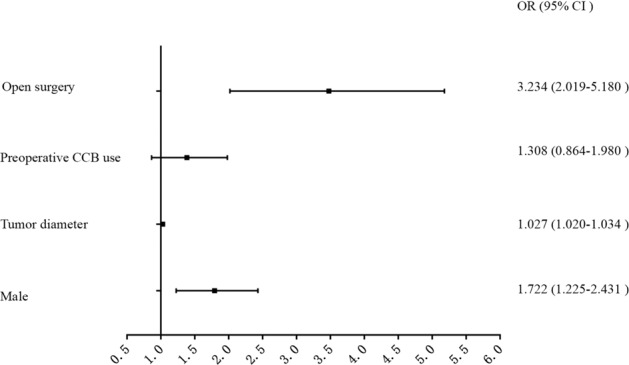
OR values of the risk factors. Abbreviation: OR odd ratio, CI confidence interval, CCB calcium channel blockade

**Fig. 2:**
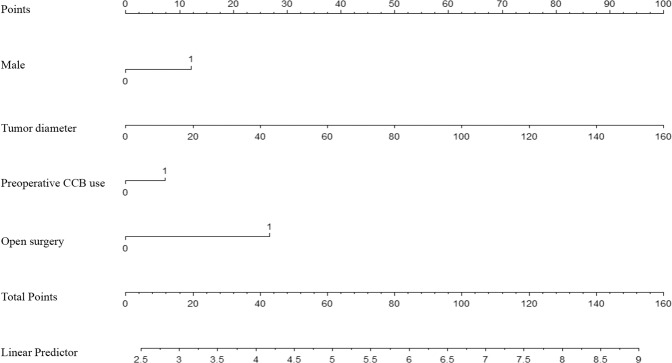
Assessment nomogram of intraoperative hemorrhage. Abbreviation: CCB calcium channel blockade

Multivariate linear regression models were constructed, and the fit of the models was assessed through AIC. Lower AIC values indicated better fit (where AIC = 157.91). The final model incorporated male gender (*β* = 0.533, OR = 1.722, *P* = 0.002), tumor diameter (*β* = 0.027, OR = 1.027, *P* < 0.001), preoperative CCB use (*β* = 0.318, OR = 1.308, *P* = 0.123) and open surgery (*β* = 1.175, OR = 3.234, *P* < 0.001) (Table 4 and Fig. 1), and a nomogram was applied (Fig. 2).

Tenfold cross validation was used for internal (*R* = 0.612, RMSE = 1.355, MAE = 1.111) and external (*R* = 0.585, RMSE = 1.398, MAE = 0.964) validation, which showed reliable results (Table 5).

**Table 5:** Validation of the assessment model

Internal validation (*N* = 251)		External validation (*N* = 120)	
*R*	0.612	*R*	0.585
RMSE	1.355	RMSE	1.398
MAE	1.111	MAE	0.964

*R* correlation coefficient, *RMSE* root mean squared error, *MAE* mean absolute error

### Comparison by gender, preoperative CCB use and surgical procedure

We made further comparisons by gender, preoperative CCB use and surgical procedure, as shown in Supplementary Table 3. Female patients were older (48.49 ± 13.98 vs. 41.78 ± 15.80, *P* < 0.001). Patients with larger preoperative BP fluctuations (SBP fluctuation: 37.72 ± 16.53 vs. 30.05 ± 13.38, *P* = 0.002; DBP fluctuation: 23.81 ± 11.45 vs. 20.34 ± 8.33, *P* = 0.035) and higher preinduction BPs (SBP: 137.47 ± 17.54 vs. 127.18 ± 18.31, *P* < 0.001; DBP: 86.60 ± 12.56 vs. 77.38 ± 12.31, *P* < 0.001) were prescribed CCBs more often. Meanwhile, these patients more often showed increases in catecholamine levels (94.83% vs. 79.27%, *P* = 0.021). Tumors in the open surgery group were larger than those in the laparoscopic adrenalectomy group (72.25 ± 35.45 vs. 47.90 ± 22.37, *P* < 0.001), but elevated catecholamine levels were more prevalent in the laparoscopic adrenalectomy group (85.44% vs. 71.11%, *P* < 0.001).

## Discussion

Our results indicate that more intraoperative hemorrhage is correlated with longer postoperative hospitalization time and more severe postoperative complications, which is consistent with existing studies [13]. Yet now there are few studies on preoperative risk factors associated with hemorrhage in PCC surgery. Current study reveals that male gender, larger tumor size, preoperative CCB use and open surgery are risk factors for more intraoperative hemorrhage in PCC surgery.

Interestingly, our study observed that males tended to suffer more intraoperative hemorrhage. We speculated that this came from gender differences in hemodynamic characteristics and from decreased vascular elasticity. Hemostasis results from vasoconstriction, while adrenergic reaction and vascular elasticity contribute to vasoconstriction [14]. Hart et al. noticed that women exhibited greater adrenergic-mediated vascular responses than men [15], and others suggested that estrogens had a preventive effect on arterial stiffness [16–21]. Although a deeper discussion is beyond the scope of this article, the leading supposition is that estrogens can protect arteries from elastin fragmentation and collagen accumulation, and the latter two lead to an increase in arterial stiffness [18–20]. Thus, overall, the influence of male gender on intraoperative hemorrhage might be due to limited vasoconstriction, which inhibits hemostasis.

In our center, CCB is applied to patients with poor BP control according to the guidelines [11]. Unexpectedly, preoperative use of CCBs increased the risk of intraoperative hemorrhage in the present study. It has been well established that CCBs, besides their antihypertensive effect, can inhibit the hemostatic process by blocking platelet activation and aggregation [22–25]. It was also found that CCBs could activate fibrinolysis, which were capable to initiate dissolution of thrombi [22, 26]. In accordance with our results, for patients taking CCBs, it may be necessary to monitor hemostatic function and titrate preoperative CCBs to avoid intraoperative hemorrhage.

Larger PCCs appeared to bleed more during the surgery in our study. Natkaniec et al. observed that compared with other adrenal tumors, PCC bled more during surgery [5], which might be due to the denser vascular network associated with increased angiogenesis [27]. Catecholamine-induced tumor angiogenesis has become an extensively studied processes recently [28]. It is important to mention that a larger PCC may produce more catecholamines, which are well-known upregulators of proangiogenic factors such as vascular permeability factor/vascular endothelial growth factor (VPF/VEGF) [28, 29]. Therefore, PCC size showed a potent impact on intraoperative hemorrhage volume. Unfortunately, methods of catecholamine detection have changed considerably over time, which makes it impossible to accurately evaluate the influence of catecholamine levels on intraoperative hemorrhage.

It was not surprising that open surgery led to more intraoperative hemorrhage. A potential explanation of our result could be the advantage of the laparoscopic procedure itself, which had been corroborated by innumerable studies [30–33]. Most studies reported that the average hemorrhage volume in laparoscopic adrenalectomy was 48–150 ml, while the average hemorrhage volume in open PCC surgery was 164–500 ml [32, 33]. An alternative factor that might help explain the result was the selection bias in tumor size. Laparoscopic adrenalectomy is the recommended approach for most PCC operations, but for PCCs larger than 6 cm, open resection is preferable to ensure complete tumor resection [11]. However, collinearity assessment ruled out this possibility.

The limitations of our study, including the retrospective design and the lack of consideration of genomic characteristics, should be taken into account. In recent years, a growing amount research on PCC gene mutations, such as VHL and SDH [34–38], has been done. Recently, the relationship between genomics and angiogenesis/metastasis has emanated in PCC studies. However, such data were not available for those diagnosed in the earlier years, and some patients declined testing. Besides, patients were included in the study at an interval of 20 years. Eventhough surgical experience does not show an impact on intraoperative hemorrhage volume in current study, the effect cannot be quantified accurately to date. In our center, PCC surgeries are performed by an experienced surgical team, which might be helpful in minimizing the amount of intraoperative hemorrhage [39, 40]. Accordingly, randomized controlled trials are needed to confirm their correlation. In addition, preoperative adrenergic blockade treatment did not show a strong association with intraoperative hemorrhage in our study, which did not imply that preoperative pharmacological management was unimportant [41, 42]. Only 6.4% of the patients skipped the preparation with PBZ, and this small sample size might have masked the benefit of PBZ.

## Conclusion

In conclusion, more intraoperative hemorrhage is correlated with longer postoperative hospitalization time and more severe postoperative complications. Our results identifies that male gender, larger tumor size, preoperative CCB use and open surgery are preoperative risk factors for hemorrhage in PCC surgery. These findings may be used to facilitate the preoperative assessment of intraoperative hemorrhage and aid in improving the individualized preoperative regimens of PCC surgery. Nevertheless, further studies are necessary to the predictive value of these variables and to investigate the precise mechanisms behind them.

## Supplementary information

Supplementary Table 1

Supplementary Table 2

Supplementary Table 3

## Data Availability

The data that support the findings of this study are available on request from the corresponding author. The data are not publicly available due to privacy or ethical restrictions.
