# Supplementary material for: Hemorrhage in pheochromocytoma surgery: evaluation of preoperative risk factors
Source: Endocrine. 2022 Apr 15;76(2):426–33. doi: 10.1007/s12020-021-02964-y (PMC9068676; doi:10.1007/s12020-021-02964-y)
Supplement: Supplementary file 1 — Supplementary Table 1 [file 12020_2021_2964_MOESM1_ESM.docx]

| **Supplementary Table 1. Relationship between estimated intraoperative hemorrhage and postoperative outcomes** | | |
| --- | --- | --- |
| Variable | Correlation analysis | |
|  | R | *P*-value |
| Postoperative Hospitalization Time | 0.454 | <0.001 |
| Clavien-Dindo Grades | 0.664 | <0.001 |
| Abbreviations: R, correlation coefficient. | | |
