# Supplementary material for: Hemorrhage in pheochromocytoma surgery: evaluation of preoperative risk factors
Source: Endocrine. 2022 Apr 15;76(2):426–33. doi: 10.1007/s12020-021-02964-y (PMC9068676; doi:10.1007/s12020-021-02964-y)
Supplement: Supplementary file 2 — Supplementary Table 2 [file 12020_2021_2964_MOESM2_ESM.docx]

| **Supplementary Table 2. Features excluded by linear regression analysis and AIC** | | | |
| --- | --- | --- | --- |
| Variables | β | t-value | *P*-value |
| Age | -0.078 | -1.232 | 0.219 |
| Family History | 0.039 | 0.609 | 0.543 |
| BMI | 0.091 | 1.436 | 0.152 |
| Hypertension | -0.043 | -0.682 | 0.496 |
| Pathoglycemia | 0.034 | 0.542 | 0.589 |
| RBC Count | 0.080 | 1.259 | 0.209 |
| Hb | 0.070 | 1.108 | 0.269 |
| HCT | 0.057 | 0.901 | 0.369 |
| ALB | 0.103 | 1.629 | 0.105 |
| Elevated Catecholamines | 0.059 | 0.935 | 0.351 |
| PBZ Use | 0.030 | 0.476 | 0.635 |
| PBZ Treatment Duration | 0.062 | 0.985 | 0.326 |
| β-adrenoceptor Blocker Use | 0.152 | 2.431 | 0.016 |
| β-adrenoceptor Blocker Treatment Duration | 0.109 | 1.726 | 0.086 |
| CCB Treatment Duration | 0.047 | 0.738 | 0.462 |
| Preoperative SBP Fluctuation | 0.171 | 2.741 | 0.007 |
| Preoperative DBP Fluctuation | -0.049 | -0.772 | 0.441 |
| Preinduction SBP | 0.038 | 0.604 | 0.546 |
| Preinduction DBP | 0.007 | 0.113 | 0.910 |
| Preinduction HR | 0.084 | 1.325 | 0.187 |
| ASA score | 0.065 | 1.020 | 0.309 |
| Abbreviations: AIC, Akaike information criterion; BMI, body mass index; RBC, red blood cell; Hb, hemoglobin; HCT, hematocrit; ALB, albumin; PBZ, phenoxybenzamine; CCB, calcium channel blocker; SBP, systolic blood pressure; DBP, diastolic blood pressure; HR, heart rate; ASA, American Society of Anesthesiologists. | | | |
