# Supplementary material for: Hemorrhage in pheochromocytoma surgery: evaluation of preoperative risk factors
Source: Endocrine. 2022 Apr 15;76(2):426–33. doi: 10.1007/s12020-021-02964-y (PMC9068676; doi:10.1007/s12020-021-02964-y)
Supplement: Supplementary file 3 — Supplementary Table 3 [file 12020_2021_2964_MOESM3_ESM.docx]

| **Supplementary Table 3. Comparison by gender, preoperative CCB use and surgical procedure** | | | | | | | | | | | |
| --- | --- | --- | --- | --- | --- | --- | --- | --- | --- | --- | --- |
| Variables | Gender difference | | |  | CCB use difference | | |  | Surgical procedure | | |
|  | Female (N=137) | male (N=114) | *P*-value |  | Non-CCB use (N=193) | CCB use (N=58) | *P*-value |  | Open (N=45) | LA (N=206) | *P*-value |
| Age, years | 48.49±13.98 | 41.78±15.80 | <0.001 |  | 45.68±15.46 | 44.64±14.28 | 0.646 |  | 45.22±13.86 | 45.49±15.48 | 0.915 |
| Elevated Catecholamines, N/% | 126, 91.97% | 109, 95.61% | 0.239 |  | 153, 79.27% | 55, 94.83% | 0.021 |  | 32, 71.11% | 176, 85.44% | <0.001 |
| Tumor Diametor, mm | 52.94±26.51 | 51.46±27.29 | 0.666 |  | 51.27±26.23 | 55.58±28.69 | 0.284 |  | 72.25±35.45 | 47.90±22.37 | <0.001 |
| Preoperative SBP Fluctuation, mmHg | 32.35±13.84 | 31.19±15.31 | 0.530 |  | 30.05±13.38 | 37.72±16.53 | 0.002 |  | 34.69±15.80 | 31.20±14.17 | 0.144 |
| Preoperative DBP Fluctuation, mmHg | 21.93±9.79 | 20.18±8.48 | 0.135 |  | 20.34±8.33 | 23.81±11.45 | 0.035 |  | 22.56±10.03 | 20.83±9.05 | 0.257 |
| Preinduction SBP, mmHg | 130.46±19.82 | 128.47±17.08 | 0.401 |  | 127.18±18.31 | 137.47±17.54 | <0.001 |  | 132.47±21.46 | 128.92±17.93 | 0.248 |
| Preinduction DBP, mmHg | 79.92±13.24 | 79.02±12.62 | 0.583 |  | 77.38±12.31 | 86.60±12.56 | <0.001 |  | 82.18±15.39 | 78.93±12.31 | 0.127 |
| Abbreviations: CCB, calcium channel blocker; LA, laparoscope adrenalectomy; BMI, body mass index; PBZ, phenoxybenzamine; SBP, systolic blood pressure; DBP, diastolic blood pressure; HR, heart rate. | | | | | | | | | | | |
